# Case report: dynamic personalized physiological monitoring in lung cancer using wearable data

**DOI:** 10.3389/fonc.2024.1420888

**Published:** 2024-10-04

**Authors:** Joshua W. Bliss, Whitney P. Underwood, Adele M. Carlson, Jessica M. Scott, Robert Daly, Bob T. Li, Alexander Drilon, Peter Stetson, Paul C. Boutros, Lee W. Jones

**Affiliations:** ^1^ New York Presbyterian, Weill Cornell Medicine, New York, NY, United States; ^2^ Department of Medicine, Division of Solid Tumor Oncology, Memorial Sloan Kettering Cancer Center, New York, NY, United States; ^3^ Department of Human Genetics, University of California, Los Angeles, Los Angeles, CA, United States; ^4^ Department of Medical Biophysics, University of Toronto, Toronto, ON, Canada; ^5^ Institute for Precision Health, University of California, Los Angeles, Los Angeles, CA, United States; ^6^ Jonsson Comprehensive Cancer Center, University of California, Los Angeles, Los Angeles, CA, United States

**Keywords:** cancer, wearable sensors, physiologic monitoring, functional status, lung cancer, exercise, prevention

## Abstract

Pretreatment prognostication, on-treatment monitoring, and early detection of physiological symptoms are considerable challenges in cancer. We describe the feasibility of high-resolution wearable data (steps per day, walking speed) to longitudinally profile physiological trajectories extracted from Apple Health data in three patients with lung cancer from diagnosis through cancer treatment after obtaining informed consent. We used descriptive statistics to describe our approach of building longitudinal physiological profiles. The wearable data monitoring period ranged from 58 to 135 weeks, with between 34,319 and 103,535 distinct digital physiological measures collected during this period—the equivalent to 41 measures per day/patient. Longitudinal profiling revealed that wearable data accurately captured physiological changes linked with clinical events such as surgery and hospitalizations as well as initiation (and cessation) of systemic cancer treatment in all three patients. These findings suggest that wearable devices could play a critical role in the management of lung cancer, although larger studies are needed to confirm these preliminary observations and validate their generalizability. Wearable devices hold significant promise for the development of personalized “digital biomarkers,” which may enhance risk stratification and management in oncology.

## Introduction

One of the most frequently asked questions a patient will ask an oncologist upon receiving a diagnosis of cancer is: “how debilitating will my treatments be?” In contemporary practice, pretreatment measures (e.g., performance status, age, cardiac echocardiography) are used to evaluate patient appropriateness and expected physical tolerability of the indicated treatment regimen, followed by on-treatment symptom monitoring using a diverse combination of tools (e.g., performance status, blood panels) with consideration relative to pretreatment or recent assessment data (1). However, pretreatment prognostication and on-treatment early detection of physiological symptoms is a considerable challenge due to interpatient heterogeneity in the nature, magnitude, and progression of symptoms (2). Improved prediction and monitoring of symptoms and the development of new biomarkers are limited by the cost and inconvenience of in-person clinical assessments. Additionally, low sampling frequency due to periodic intervals of in-person visits during the treatment course further limits monitoring. Failure to detect early physiological symptoms contributes to suboptimal therapeutic management and poor clinical outcomes (3, 4). Wearable data collected by devices such as mobile smartphones and smartwatches with integrated multisensory systems generate hundreds of unique, quantitative measures of patient physiology/lifestyle at different phenotypic levels (e.g., mobility and function) (5). These devices are able to monitor metrics of interest on a near-continuous basis (i.e., high-frequency sampling) over long periods of time, with all data captured passively without patient manual input. The use of wearables and other digital health devices show promising utility to longitudinally capture physiologic and lifestyle data in several clinical settings (5–8), but their application in cancer is limited (9). Here, we report the feasibility of wearable data (in this case, Apple iPhone^®^ with HealthKit^®^) to perform high-resolution, personalized mapping of physiological trajectories in three patients with lung cancer.

## Methods

### Patient recruitment

All study procedures were reviewed and approved by the Memorial Sloan Kettering Cancer Center (MSK) institutional review board (NCT05390827). Inclusion criteria included patients ≥18 years of age with stage III or IV lung cancer with access to an Apple iPhone^®^ with Apple Health functionality. Exclusion criteria included stage I or II lung cancer, no access to an Apple iPhone^®^ and Apple Health data that did not include at least 6 months prior and 1 year following the date of cancer diagnosis. Eligible patients were identified and approached by a member of the patient’s care team or study personnel at Memorial Sloan Kettering Cancer Center (MSK) during a routine in-person clinical visit. If interested, patients viewed a ~60-s video overviewing the study purpose, rationale, and procedures. Appropriate patients provided *e*consent and then were automatically redirected to video instructions on how to extract and share Apple Health data from their mobile device. Investigators obtained informed consent to publish information from all patients.

### Data collection and statistics

**Figure 1:**
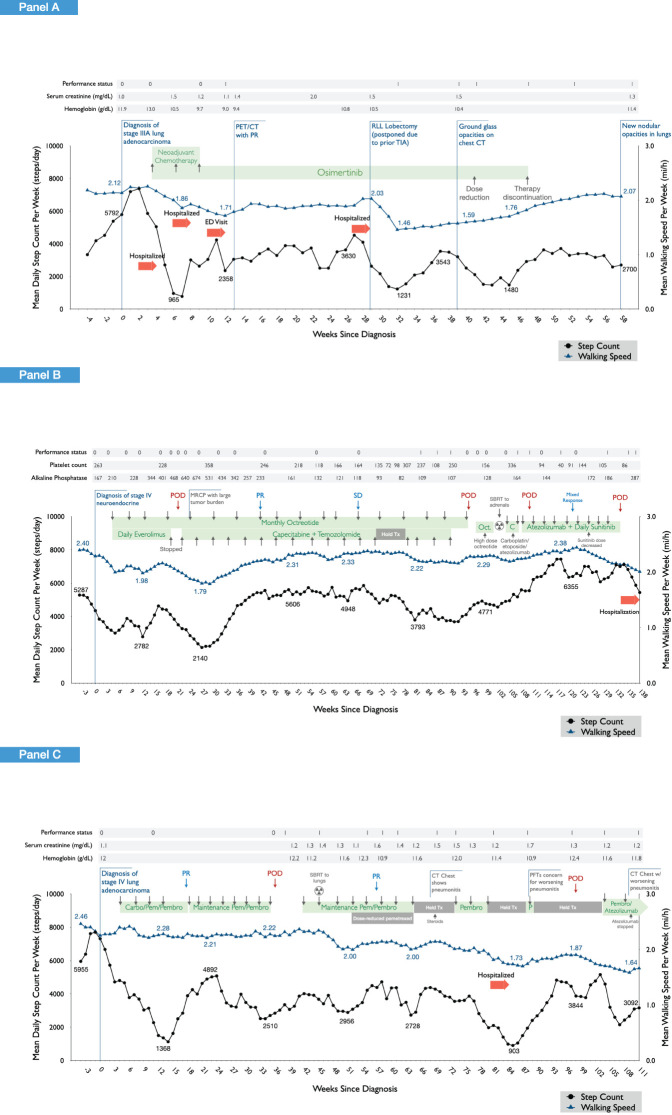
Wearable physiological data for the three study patients across the cancer treatment continuum. Longitudinal profiling of daily step count and walking speed (moving average at 4-week intervals) are shown in relation to clinical events observed in each of the three patients. Panel **(A)** shows the findings in patient 1, a 64-year-old woman, diagnosed with T1bN2M0 (stage IIIA) lung adenocarcinoma and treated with neoadjuvant cisplatin, pemetrexed, and osimertinib followed by a right lower lobe lobectomy and mediastinal lymph node dissection with an uncomplicated postoperative course. After surgery, daily osimertinib was dose reduced due to fatigue, anorexia, and skin/nail toxicity, and subsequently discontinued. Panel **(B)** shows the findings in patient 2, a 55-year-old woman, diagnosed with stage IV neuroendocrine/carcinoid lung cancer, initially treated with daily everolimus plus monthly intramuscular octreotide. Following confirmation of progressive disease, systemic therapy was discontinued, and stereotactic body radiation therapy (SBRT) was administered followed by initiation of carboplatin, etoposide, and atezolizumab. Following confirmation of further disease progression, the patient initiated the combination of capecitabine and temozolomide. Panel **(C)** shows the findings of patient 3, a 71-year-old woman diagnosed with stage IV lung adenocarcinoma, initially treated with the combination of carboplatin, pemetrexed, and pembrolizumab. Following partial response, she transitioned to maintenance pemetrexed and pembrolizumab. She received SBRT to pulmonary metastases with subsequent onset of pneumonitis and exertional dyspnea. Following progressive disease, atezolizumab plus bevacizumab was initiated. Due to ongoing pneumonitis, atezolizumab was discontinued with continuance of bevacizumab.

Physiological data types that were extracted from Apple Health included mobility (step count), walking and running distance, walking speed, step length, and double support time (i.e., time spent with both feet on the ground during ambulation). These are standard metrics collected in Apple Health via iOS v.13 or later and have been validated for accuracy in the literature (10). For this proof-of-concept analysis, Apple Health data were exported directly from patient devices via comma separated values (.csv) files natively generated by Apple Health using the export function and directly transferred to an encrypted flash storage device. This information was deidentified and stored in encrypted institutional servers. The physiological metrics of interest were mobility [steps per day (steps/day)], a measure of physical activity, and walking speed [miles per hour (mph/h)], a measure of physical function. Physical activity and walking speed have prognostic importance in patients with cancer (11, 12). Physiological data were processed and transformed into the appropriate format using RStudio (earlier version later followed by version 2024.04.2). All medical and demographic data for the period covering wearable data extraction for each patient such as cancer-directed therapy, laboratory data, and clinical events were programmatically extracted from the MSK electronic medical record into a wearable digital chronicle mapping specific database using institution-specific extraction solutions in collaboration with MSK bioinformatics. Anonymized identification numbers were assigned to included patients and patient-specific identifiers were removed, ensuring that study involvement did not impose additional risks or burdens on participating patients. R-script and additional extraction details are available upon request. Physiological and clinical data were aggregated and overlaid onto patient-specific chronological maps for visualization (**Figure 1**). Given the primary aim of this proof-of-concept study was to manifest the feasibility of physiological and clinical data aggregation for visualization of trends over time, statistical analyses were not performed as a part of this study.

## Results

### Patient clinical cases

Patient 1 (**Figure 1A**) was a 64-year-old woman, diagnosed with T1bN2M0 (stage IIIA) lung adenocarcinoma and treated with neoadjuvant cisplatin, pemetrexed, and osimertinib followed by a right lower lobe lobectomy and mediastinal lymph node dissection with an uncomplicated post-operative course. After surgery, daily osimertinib was dose reduced due to fatigue, anorexia, and skin/nail toxicity and subsequently discontinued.

Patient 2 (**Figure 1B**) was a 55-year-old woman, diagnosed with stage IV neuroendocrine/carcinoid lung cancer, initially treated with daily everolimus plus monthly intramuscular octreotide. Everolimus was subsequently discontinued due to abnormal blood laboratory values followed by clinical progression. She then initiated capecitabine plus temozolomide leading to partial response. Upon further confirmation of progressive disease, systemic therapy was discontinued, and stereotactic body radiation therapy (SBRT) was administered followed by initiation of carboplatin, etoposide, and atezolizumab. Following confirmation of further disease progression, the patient initiated the combination of capecitabine and temozolomide.

Patient 3 (**Figure 1C**) was a 71-year-old woman diagnosed with stage IV lung adenocarcinoma, initially treated with the combination of carboplatin, pemetrexed, and pembrolizumab. Following partial response, she transitioned to maintenance pemetrexed and pembrolizumab. She received SBRT to pulmonary metastases with subsequent onset of pneumonitis and exertional dyspnea. Following progressive disease, atezolizumab plus bevacizumab was initiated. Due to ongoing pneumonitis, atezolizumab was discontinued with continuance of bevacizumab.

### Physiological data collection

**Figure 1** shows longitudinal profiling of wearable data for the three patients from 4 weeks prior to cancer diagnosis (pretreatment) and across the course of cancer treatment. Pertinent data pertaining to patient demographics, medical history, and cancer treatments are provided in the **Figure 1** legend.

Patient 1’s wearable data is presented from 24/01/2022 to 06//04/2023, a monitoring period of 438 days (**Figure 1A**). During this period, a total number of 16,051 distinct step measures and 4,149 walking speed measures were extracted (total 20,200 distinct measures), a mean of 46 distinct measures per day of monitoring. For patient 2, wearable data are presented from 06/10/2020 to 07/07/2023, a monitoring period of 1,005 days (**Figure 1B**). During this period, a total number of 25,779 distinct step measures and 34,664 walking speed measures were extracted (total 60,443 distinct measures), a mean of 60 distinct measures per day of monitoring. Patient 3’s wearable data are presented from 27/04/2021 to 21/07/2023, a monitoring period of 816 days (**Figure 1C**). During this period, a total number of 16,808 distinct step measures and 10,247 walking speed measures were extracted (total 27,055 distinct measures), a mean of 33 distinct measures per day of monitoring.

In a 2-week period prior to diagnosis, patient 1 walked a mean of 6,831 steps per day, considered within the normal range (80th percentile for a 64-year-old woman) (13). In contrast, patients 2 and 3 walked a mean of 3,564 and 12,020 steps/day, respectively. Corresponding mean walking speed data during this pre-diagnosis period were as follows: patient 1, 2.17 mph, patient 2, 2.27 mph, and patient 3, 2.32 mph. Hence, physiological status as measured by digital metrics displayed inter-patient variability despite similarities in age and cancer histology and was relatively stable in the weeks prior to diagnosis.

### Physiological alterations in response to cancer events

Longitudinal profiling revealed that wearable digital data accurately captured expected physiological changes related with cancer-related events or perturbations in all three patients. This was especially evident for overt clinical events such as surgery and hospitalizations as well as administration (and cessation) of systemic cancer treatment. For instance, in patient 1, lung surgical resection (lobectomy) linked with a substantial decrease in both daily step count and walking speed, reaching a nadir of 1,231 steps/day and 1.46 mph, respectively, ~3 weeks after surgery. Wearable data indicated a recovery period of ~9 weeks, although daily step count and walking speed did not recover to presurgical values. During this period, ECOG PS, serum creatine, and hemoglobin levels remained stable. Similar acute marked reductions in physiological metrics were observed during periods of hospitalizations in patients 1 and 3. During periods of systemic anticancer treatment, marked and sustained decreases in daily steps and walking speed were observed following administration of neoadjuvant chemotherapy for patient 1. The ECOG PS score did not change from 0; however, worsening of serum creatine and hemoglobin levels was observed during this period. Similar patterns were observed during first-line combination chemotherapy and immunotherapy in patient 3. Interestingly, comparable worsening of physiological metrics was not observed, in general, in patient 2 with first-line combination treatment with everolimus and octreotide and subsequent second-line therapy with capecitabine and temozolomide.

### Cancer event foreshadowing

In addition to accurate mapping of overt clinical perturbations, several unexpected events were also captured. For example, in patient 1, daily step count decreased from 5,503 steps/day 3 weeks prior to diagnosis to 970 steps/day (mean/week) 3 weeks after diagnosis, a period in which the individual was not receiving any form of anticancer therapy. A similar magnitude of deterioration was observed in patient 2 (6,368 to 1,763 steps/day) and patient 3 (7,626 to 3,713 steps/day) over the same monitoring period. In some instances, wearable data also linked with disease response to cancer therapy. In patient 3, a marked increase in daily steps was observed from 1,559 steps/day at week ~14 to 3,384 steps/day at week ~24, which corresponded with partial disease response documented at week 18. In patient 2, disease progression at week ~19 was followed by a steady decline in daily steps (and walking speed), reaching a nadir of 1,974 steps/day at week 26 compared with 4,671 steps/day at week ~16, linked with extensive tumor burden. Physiological metrics subsequently improved substantially, corresponding with initiation of new systemic therapy and subsequent partial disease response at week ~41.

Finally, longitudinal profiling revealed several instances in which changes in wearable data preceded overt clinical events. For example, in patient 1, marked and progressive decreases in daily steps were observed from the point just prior to neoadjuvant chemotherapy initiation (7,539 steps/day) that preceded a hospitalization at week 4 (2,204 steps/day). Also in this patient, progressive decreases in daily steps were observed ~6 weeks prior to discontinuation of single-agent osimertinib at week 47 in the setting of progressively worsening fatigue, anorexia, and skin/nail toxicities. In patient 2, progressive decreases in daily steps were observed starting at week 16 (4,671 steps/day), preceding the diagnosis of progressive disease at week 21 (3,030 steps/day). Nevertheless, in this patient progressive increases in step count were observed from week 91 (3,688 steps/day) to week 118 (6,286 steps/day), yet two instances of disease progression were observed during monitoring period.

## Discussion

This proof-of-concept study showed that widely available, low-cost data collected via a wearable device (i.e., smartphone) provide accurate and reliable objective measures of patient physiology among patients with cancer. Use of two standard basic “digital biomarkers” (daily steps and walking speed) revealed considerable variation in patient status at diagnosis, despite similar diagnoses and “good” ECOG performance. Wearable data may therefore have promise to provide highly discriminatory, personalized baselines to augment pretreatment prognostication and longitudinal detection of deviations from baseline during the treatment course. Indeed, longitudinal profiling of daily steps accurately captured both the acute and recovery trajectory of diverse cancer-related events or perturbations (e.g., initiation and cessation of cancer therapy, serious adverse events) with responses being highly patient specific (i.e., a digital physiologic profile of the individual). The availability of several physiological metrics per standard on most smartphones also enables evaluation of multiple orthogonal biomarkers simultaneously, such as analysis of walking speed alongside daily steps in the present study which may provide distinct but complementary information to further enhance prognostication.

Most cancer patients attend visits with the oncology team every ~4 weeks during active treatment and every 6 to 12 months after therapy. Longitudinal physiological mapping at high resolution may contribute to “filling in the gaps” on the status and well-being of a patient in between clinical visits. Symptom monitoring using electronic patient-reported outcomes (*e*PROs) in conjunction with appropriate symptom management improves clinical outcomes (14); however, these tools require manual patient entry, which limits the frequency of symptom assessment and lowers patient engagement over the long term. Leveraging of a wearable device enabled near continuous profiling of patient physiological status/symptoms across almost the entirety of the cancer treatment continuum, revealing highly personalized patterns of response. Intriguingly, our initial data suggest that continuous monitoring may also permit mapping and perhaps even early detection of serious clinical events. As wearable devices become more accessible and ubiquitous across patient populations, their use in clinical research is also expanding (15) and is showing accuracy comparable with clinical devices and manual measurements (16, 17).

It is important to recognize the limitations of this proof-of-concept study, which include a small sample size (three patients), the lack of statistical analyses for data relation significance, inter-patient variability in time spent wearing the device, and the potential biases involved with including only patients with access to Apple devices, which could lead to innate confounders. Future work by our group will involve project expansion to include more patients in future studies, the integration of Apple Health and the MSK electronic medical record for streamlined data extraction, expansion of physiological metrics such as sleep data and heartrate variability, statistical analyses of a larger study cohort to assess for clinically significant associations between physiological signals and clinical outcomes, and the use of machine learning for predictive algorithm development. We opted for the use of Apple devices given the uniformity with which data are collected and extracted via Apple Health functions. Future directions include expansion into other devices to address potential biases.

Overall, this study supports the provocative hypothesis that high-resolution wearable data may detect subtle but important physiological changes not captured by current subjective and less discriminatory tools such as PS scoring tools, leading to improved clinical outcomes (18). In conclusion, high-resolution, longitudinal objective physiological monitoring via wearable technology holds significant promise for the development and validation of personalized “digital biomarkers”. Such biomarkers may augment oncology risk stratification and management.

## Data Availability

The raw data supporting the conclusions of this article will be made available by the authors, without undue reservation.
